# Evaluation of Diagnostic Methods and Rifampicin Resistance in Pulmonary Tuberculosis: A Hospital-Based Study

**DOI:** 10.7759/cureus.67062

**Published:** 2024-08-17

**Authors:** Priyanka Joshi, Krishna G Singh, Vishal Patidar, Vikas Gupta

**Affiliations:** 1 Pulmonary Medicine, Mahaveer Institute of Medical Sciences and Research, Bhopal, IND; 2 Respiratory Medicine, Chirayu Medical College and Hospital, Bhopal, IND; 3 Respiratory Medicine, Amaltas Institute of Medical Sciences, Bangar Dewas, IND; 4 Community Medicine, Government Medical College, Seoni, IND

**Keywords:** sensitivity, drug-resistant tuberculosis, rifampicin resistance, smear afb examination, cbnaat

## Abstract

Background

Tuberculosis (TB) is an infectious disease caused by Mycobacterium tuberculosis, predominantly affecting the lungs (pulmonary TB) and is a significant public health challenge in India. The study aims to analyze demographic, radiological, and clinical subgroups of pulmonary TB cases, examine the relationship between smear acid-fast bacillus (AFB examination) and cartridge-based nucleic acid amplification test (CBNAAT), evaluate CBNAAT sensitivity for Mycobacterium tuberculosis (MTB) in new and previously treated patients, and determine the proportion of rifampicin resistance.

Methods

This hospital-based prospective study was conducted among patients diagnosed with pulmonary TB at the Respiratory Medicine Department of a Government Hospital over 16 months (August 2019 to December 2020). The study included 150 diagnosed TB cases (new and previously treated). Data collection encompassed demographic details, clinical symptoms, comorbidities, radiological findings (chest X-ray), and microbiological results (smear AFB examination, CBNAAT). Sputum samples were subjected to Ziehl-Neelsen staining and CBNAAT for MTB detection and rifampicin resistance testing. Statistical analysis was performed using IBM SPSS Statistics version 21.0 (IBM Corp., Armonk, NY, USA).

Results

Of the 150 patients, 69.3% were male, and 48% were aged 21-40 years. The majority had a BMI of 18.5-24.9 kg/m² (50%) and resided in urban areas (63.3%). Common symptoms included cough (95.3%), fever (80%), and weight loss (74%). Cavitary lesions on chest X-ray were observed in 84% of patients. Smear microscopy detected MTB in 72.7% of cases, while CBNAAT detected MTB in 94% of cases. CBNAAT sensitivity for smear-positive and smear-negative samples was 93.97% and 94.12%, respectively. Rifampicin resistance was found in 3% of new cases and 6% of previously treated cases. The sensitivity of smear microscopy was 77.33%, and the sensitivity of CBNAAT was 94%.

Conclusion

The study underscores the high burden of pulmonary TB and the utility of CBNAAT in detecting MTB and rifampicin resistance, particularly in smear-negative samples. The findings highlight the necessity of universal drug susceptibility testing (DST) for effective TB management and the importance of addressing drug resistance to improve treatment outcomes.

## Introduction

Tuberculosis (TB) is an infectious disease caused by Mycobacterium tuberculosis (MTB), transmitted mostly via droplet nuclei. There are mainly two clinical forms, pulmonary tuberculosis (PTB), and extrapulmonary tuberculosis. India is the country with the highest burden of tuberculosis. In 2019, there were an estimated 10 million new (incident) TB cases globally, of which 56% were among men, 32% were among women, and 12% were among children [1,2]. People living with HIV accounted for 8.2% of total cases [3].

In India, the incidence of TB was estimated to be 2.64 million (193 per 100,000 population). Globally, India accounted for 26% of total HIV-TB co-infected patients, with 2.7% among incident TB cases [4]. In 2019, an estimated 465,000 people had multidrug-resistant TB (MDR-TB) infection worldwide, and India accounted for 27% of the total burden [5]. An estimated 3.3% of new TB cases and 18% of previously treated cases worldwide had MDR-TB [6]. In India, the incidence of MDR-TB was 2.8% among new cases and 14% among previously treated cases [6].

The World Health Organization (WHO) defines universal access to drug susceptibility testing (DST) as rapid DST for at least rifampicin, and further DST for at least fluoroquinolones and second-line injectable agents in all TB patients with rifampicin resistance. Drug-resistant tuberculosis (DR-TB) poses a major threat to the control of tuberculosis worldwide [7]. The program is currently scaling up its policy of universal DST, whereby all cases diagnosed with TB will receive a minimum of rifampicin resistance testing, categorizing into two subtypes, rifampicin sensitive and rifampicin resistant [8].

In settings of high MDR-TB (MDR-TB rate > 5% among new cases and > 20% among re-treatment cases), a cartridge-based nucleic acid amplification test (CBNAAT) is performed to rule out rifampicin resistance before the initiation of treatment [9].

So, the present study aimed to analyze the demographic, clinical, radiological, and microbiological characteristics of PTB patients, with a focus on the sensitivity of CBNAAT (GeneXpert; Cepheid, Sunnyvale, CA, USA) and the prevalence of rifampicin resistance.

## Materials and methods

Study design

This study was a hospital-based prospective study conducted among patients diagnosed with PTB pending treatment in the Respiratory Medicine Department of Government Hospital over 16 months, from August 2019 to December 2020.

Study population

The study enrolled a total of 150 diagnosed PTB cases, including new and previously treated cases where treatment had not yet been initiated. Exclusion criteria included patients who were unwilling to participate or refused consent, those incapable of giving consent due to psychiatric illness, individuals with extrapulmonary tuberculosis, pregnant women, and patients with disseminated tuberculosis.

Data collection

Data was prospectively collected through structured interviews, clinical examinations, medical record reviews, and laboratory test results. Blood tests included complete blood count, liver function tests, renal function tests, and random blood sugar levels. Demographic information included age, sex, occupation, locality, socioeconomic class, height, weight, BMI, and family history of PTB. Clinical data encompassed symptoms and duration of illness (cough, expectoration, fever, dyspnea, hemoptysis, and chest pain), and comorbidities. Radiological assessments included chest X-ray findings detailing the type and extent of lesions. Microbiological data were gathered through smear acid-fast bacilli (AFB) examination and results from CBNAAT.

Diagnostic procedures

Sputum samples were collected at diagnosis and subjected to Ziehl-Neelsen staining for AFB detection. AFB presence was graded from "Scanty" to "+1," "+2," and "+3." Sputum samples for CBNAAT testing (GeneXpert) were analyzed for MTB, with results categorized as "MTB Detected," "Not Detected," "Invalid," and "Indeterminate." Universal drug sensitivity test screening for rifampicin was performed through CBNAAT at DTC, categorizing samples as rifampicin-sensitive or rifampicin-resistant (with or without resistance to other anti-TB drugs).

Patients were monitored throughout the treatment period, with follow-up visits at regular intervals to assess clinical progress, treatment adherence, and any adverse drug reactions. Additional sputum samples were collected during follow-up for repeat smear AFB examination and CBNAAT testing as needed.

Statistical analysis

Data were analyzed using SPSS software version 21.0 (IBM Corp., Armonk, NY, USA). Descriptive statistics summarized demographic, clinical, and radiological characteristics. The correlation between smear AFB examination and CBNAAT results was assessed using Fisher's exact test. Sensitivity and specificity of CBNAAT were calculated. The association between rifampicin resistance and independent variables (demographic, clinical, and radiological) was assessed using Fisher's exact test. A p-value < 0.05 was considered statistically significant.

Ethical considerations

The study protocol was reviewed and approved by the Institutional Ethics Committee (Approval number: MIMSR/IEC/2018/6/006). Written informed consent was obtained from all participants prior to enrollment. Confidentiality of patient information was strictly maintained throughout the study.

## Results

The study included 150 participants, predominantly male (69.3%) and aged 21-40 years (48.0%). Most had a BMI between 18.5-24.9 kg/m² (50.0%) and resided in urban areas (63.3%). Over half had addictions (54.7%), mainly tobacco chewing (30.7%). The majority belonged to the low socioeconomic class (78.0%) (Table 1).

**Table 1:** Baseline characteristics of the study participants.

Variables	Frequency (%)
Gender	
Male	104 (69.3)
Female	46 (30.7)
Age group	
<21 years	17 (11.4)
21-40 years	72 (48.0)
41-60 years	38 (25.3)
>60 years	23 (15.3)
Body Mass Index (kg/m^2^)	
<18.5	60 (40.0)
18.5-24.9	75 (50.0)
25.0-29.9	14 (9.3)
30.0 or more	1 (0.7)
Residence	
Rural	55 (36.7)
Urban	95 (63.3)
Addiction	82 (54.7)
Tobacco chewer	46 (30.7)
Smoker	26 (17.3)
Alcoholic	10 (6.7)
No addiction	68 (45.3)
Socioeconomic class	
Low	117 (78.0)
Middle	29 (19.3)
High	4 (2.7)

Among 150 participants, 66.7% were new TB cases and 33.3% were previously treated. The previously treated TB cases included individuals who had completed standard first-line anti-TB therapy and those who had received second-line treatment regimens due to drug resistance or treatment failure. Specifically, 20% of the previously treated cases had completed first-line therapy (n=30), while 13.3% had undergone second-line treatment (n=20). A family history of drug-sensitive TB was present in 13.3% and drug-resistant TB in 1.3%. The most common clinical symptoms were cough (95.3%), fever (80.0%), and expectoration (76.7%). Chest X-ray showed cavitary lesions in 84.0% and extensive lesions (more than one lobe) in 63.3%. Comorbidities were present in 33.3% of participants, with diabetes (8.7%), coronary artery disease (6.6%), and chronic obstructive pulmonary disease (6.0%) being the most prevalent. No comorbidities were reported in 66.7% of the participants (Table 2).

**Table 2:** Clinical profile of the study participants. TB: Tuberculosis, HIV: Human immunodeficiency virus

Variables	Frequency (%)
Treatment History of TB	
New	100 (66.7)
Previously treated	50 (33.3)
Family history of Pulmonary TB	
Drug sensitive TB	20 (13.3)
Drug resistant TB	2 (1.3)
No history	128 (85.4)
Clinical symptoms	
Cough	143 (95.3)
Expectoration	115 (76.7)
Dyspnea	33 (22.0)
Fever	120 (80.0)
Hemoptysis	17 (11.3)
Chest pain	25 (16.7)
Weight loss	111 (74.0)
Type of lesion on Chest X Ray	
Cavitary	126 (84.0)
Non cavitary	24 (16.0)
Extent of lesion on Chest X Ray	
1 lobe (Limited)	55 (36.7)
>1 lobe (Extensive)	95 (63.3)
Comorbidity	50 (33.3)
Diabetes	13 (8.7)
Coronary Artery Disease	10 (6.6)
Chronic Obstructive Pulmonary Disease	9 (6.0)
Hypertension	9 (6.0)
HIV	7 (4.6)
Carcinoma Colon	1 (0.7)
Vasculitis	1 (0.7)
No comorbidity	100 (66.7)

In this study out of 150 pulmonary tuberculosis patients, sputum smear microscopy was able to detect 116 (77.3%) cases while sputum CBNAAT detected 141 (94%) cases. Among the 150 participants, AFB grading results showed that 13.3% had scanty AFB, 23.3% had 1+, 18.7% had 2+, 22.0% had 3+, and 22.7% were negative. CBNAAT results indicated that MTB was detected in 94.0% of cases, with 90.0% being rifampicin-sensitive (Rif S) and 4.0% rifampicin-resistant (Rif R). MTB was not detected in 6.0% of cases (Table 3).

**Table 3:** Sputum smear AFB grading and CBNAAT. AFB: acid-fast bacilli, CBNAAT: cartridge-based nucleic acid amplification test, MTB: Mycobacterium tuberculosis, Rif S: rifampicin-sensitive, Rif R: rifampicin-resistant

Variables	Frequency (%)
AFB grading	
Scanty	20 (13.3)
1+	35 (23.3)
2+	28 (18.7)
3+	33 (22.0)
Negative	34 (22.7)
CBNAAT	
MTB detected	141 (94.0)
Rif S	135 (90.0)
Rif R	6 (4.0)
MTB not detected	9 (6.0)

Among the 150 participants, MTB was detected in 109 patients (72.7%) using both smear microscopy and CBNAAT. In 32 patients (21.3%), MTB was detected by CBNAAT but not by smear microscopy, whereas in seven patients (4.67%), MTB was identified by smear microscopy but not by CBNAAT. In two clinically diagnosed PTB patients (1.3%), both tests were negative. The sensitivity of CBNAAT for MTB detection in smear-positive samples was 93.9%, and in smear-negative samples, it was 94.1% (Table 4).

**Table 4:** Correlation between CBNAAT and smear microscopy. CBNAAT: cartridge-based nucleic acid amplification test, MTB: Mycobacterium tuberculosis

Variables	Smear Positive (n=116)	Smear Negative (n=34)	P value
CBNAAT MTB detected (n=141)	109	32	0.973
CBNAAT MTB not detected (n=9)	7	2	

Among the 141 participants with detected MTB, 135 were Rif S and six were Rif R. No significant differences were found between Rif S and Rif R groups in terms of gender, age, BMI, residence, comorbidity, addiction history, socioeconomic class, or previous TB history. However, family contact exposure to drug-resistant TB was significantly associated with rifampicin resistance (P = 0.004). Chest X-ray lesions were predominantly cavitary in both groups, with extensive lesions more common in the Rif R group, though not statistically significant (Table 5).

**Table 5:** Association of rifampicin sensitivity with the independent variables of the study participants (n=141). TB: Tuberculosis

Variables	Rif S (n=135)	Rif R (n=6)	P value
	Frequency (%)		
Gender			
Male (n=99)	95 (70.4)	4 (66.7)	0.846
Female (n=42)	40 (29.6)	2 (33.3)	
Age group			
60 years or less (n=119)	114 (84.4)	5 (83.3)	0.941
>60 years (n=22)	21 (15.6)	1 (16.7)	
Body Mass Index (kg/m^2^)			
<18.5 (n=53)	50 (37.0)	3 (50.0)	0.521
18.5 or more (n=88)	85 (63.0)	3 (50.0)	
Residence			
Urban (n=89)	86 (63.7)	3 (50.0)	0.496
Rural (n=52)	49 (36.3)	3 (50.0)	
Comorbidity			
Present (n=48)	56 (41.5)	2 (33.3)	0.794
Absent (n=93)	89 (65.9)	4 (66.7)	
Addiction history			
Addiction (n=79)	76 (56.3)	3 (50.0)	0.761
No addiction (n=62)	59 (43.7)	3 (50.0)	
Socioeconomic class			
Low (n=110)	106 (78.5)	4 (66.7)	0.636
Middle (n=27)	25 (18.5)	2 (33.3)	
High (n=4)	4 (3.0)	0 (0.0)	
Previous history of Pulmonary TB			
New (n=92)	89 (65.9)	3 (50.0)	0.422
Previously treated (n=49)	46 (34.1)	3 (50.0)	
Family contact exposure			
Drug Sensitive TB (n=16)	15 (11.1)	1 (16.7)	0.004
Drug Resistant TB (n=2)	1 (0.7)	1 (16.7)	
Absent (n=123)	119 (88.1)	4 (66.7)	
Chest X Ray lesion			
Cavitary (n=121)	115 (85.2)	6 (100.0)	0.308
Non cavitary (n=20)	20 (14.8)	0 (0.0)	
Chest X Ray lesion extent			
Limited (n=48)	48 (35.6)	0 (0.0)	0.072
Extensive (n=93)	87 (64.4)	6 (100.0)	

## Discussion

This hospital-based prospective study aimed to analyze the demographic, clinical, radiological, and microbiological characteristics of PTB patients, with a focus on the sensitivity of CBNAAT (GeneXpert) and the prevalence of rifampicin resistance. Our findings provide valuable insights into the current status of PTB management and highlight the efficacy of CBNAAT as a diagnostic tool.

The study population was predominantly male (69.3%), which is consistent with previous studies where males are more commonly affected than females. Kandi et al. reported a male predominance of 60%, while Subbarao et al. found 68% male participants [10,11]. Similarly, Mukherjee et al. noted 59.2% male representation, and Patel et al. reported 66.9% males in their respective studies [12,13]. Senthilnathan et al. observed 65% male participants [14]. The majority of patients were aged 21-40 years (48.0%), reflecting the higher burden of TB in economically productive age groups. A significant proportion of the patients (40.0%) had a BMI below 18.5 kg/m², indicating malnutrition, which is a known risk factor for TB. Similarly, BMI below 18.5 kg/m² was observed in around half of the study participants in the studies by Senthilnathan et al. (51.3%) and Kumarnatarajan et al. (47.6%) [14,15]. Low BMI increases TB risk due to weakened immune function from malnutrition, reduced immune cell production, altered cytokine levels, impaired mucosal barriers, and disrupted metabolic processes. These factors compromise the body's ability to fight off Mycobacterium tuberculosis, enhancing susceptibility to infection and disease progression. The majority of the patients belonged to the low socioeconomic class (78.0%), which is consistent with the higher incidence of TB in economically disadvantaged populations. Addressing social determinants of health is crucial in the fight against TB. 

Common symptoms reported were cough (95.3%), fever (80.0%), and expectoration (76.7%). Weight loss was observed in 74.0% of patients, aligning with the typical clinical presentation of PTB. Similarly, Kumarnatarajan et al. reported 100% prevalence of cough, 39% for fever, 64% for hemoptysis, and 70% for weight loss; but in contrast with the study by Datta et al., where cough was noted in 90.01%, fever in 57.7%, hemoptysis in 53%, and weight loss in 61% of PTB cases [15,16]. Comorbidities were present in 33.3% of patients, with diabetes being the most common (8.7%). The present study documented an incidence of 4.7% of HIV-TB coinfection. The incidence of HIV-TB coinfection varied across several studies. Basavaraj et al. reported a rate of 2.58%, Kandi et al. found 6%, Subbarao et al. observed 8.13%, and Mukherjee et al. noted a higher rate of 14.47% [10-12,17]. In our study, radiologically, cavitary lesions were prevalent in 84.0% of cases. This contrasts with findings from Mohanty et al., where cavitary lesions were observed in 16% of cases, Bhattacharya et al. who reported a prevalence of 40%, Panda et al. who found 23.5%, and Rout et al. who noted 37.5% [18-21].

The sensitivity of smear microscopy for the detection of MTB shows notable variability across different studies. In the present study, the sensitivity was reported at 77.33%, which is higher compared to studies by Kandi et al. (41.5%), Senthilnathan et al. (21.3%), Subbarao et al. (20.08%), Mukherjee et al. (16.7%), and Patel et al. (72%) [10-14]. These differences can be attributed to several factors, including variations in laboratory personnel expertise, differences in sample size, the quality of sputum samples collected, and methodological approaches used for diagnosis. Such variability highlights the challenges in standardizing diagnostic techniques for TB and underscores the importance of optimizing laboratory practices to improve diagnostic accuracy.

In the present study, CBNAAT demonstrated a sensitivity of 94.0%. This compares favorably with findings from Panayotis et al. (90.6%), Sharma et al. (95.7%), and Patel et al. (86.8%), however, Theron et al. reported a slightly lower sensitivity of 78.7% [3,13,22,23]. The study demonstrated that CBNAAT is highly sensitive in detecting MTB, with a sensitivity of 93.9% in smear-positive samples and 94.1% in smear-negative samples. These results underscore the utility of CBNAAT in rapid and accurate TB diagnosis, particularly in smear-negative cases where traditional microscopy may fail to detect the pathogen. Theron et al. reported a sensitivity of 94.7% in smear-positive samples and 46.8% in smear-negative samples [3]. Subbarao et al. found a sensitivity of 100% in smear-positive samples and 73% in smear-negative samples [11]. Similarly, Mukherjee et al. observed a sensitivity of 100% in smear-positive samples and 38.4% in smear-negative samples [12]. Sharma et al. reported a sensitivity of 99.2% in smear-positive samples and 77.7% in smear-negative samples [23]. Dewan et al. noted a sensitivity of 100% in smear-positive samples and 32.6% in smear-negative samples [24].

Rifampicin resistance was found in 4.0% of cases, which is a significant concern as it indicates MDR-TB. Basavaraj et al. reported a prevalence of 2.5%, Senthilnathan et al. found 3.8%, Subbarao et al. noted 2.7%, Mukherjee et al. observed 2.2%, and Patel et al. recorded 4.5% rifampicin resistance [11-14,17]. In the present study, rifampicin resistance was found in 3.0% of newly diagnosed and 6.0% of previously treated patients. According to the WHO Global Tuberculosis Report, 2020, the incidence of rifampicin resistance among newly diagnosed patients was reported as 2.8% in India, 7.1% in China, and globally 3.32% [4]. For previously treated patients, the incidence was higher at 14% in India, 23% in China, and globally 17.7% [4]. These figures highlight varying rates of rifampicin resistance across different regions and underscore the importance of regional surveillance and effective treatment strategies to combat drug-resistant tuberculosis.

The study found no significant association between rifampicin resistance and demographic variables such as gender, age, BMI, or residence. Our analysis revealed that there were no significant differences between the Rif S and Rif R groups in terms of smoking history. Additionally, we found no significant differences between these groups regarding alcohol use. However, a significant association was observed with a family history of drug-resistant TB (P = 0.004). This underscores the critical importance of contact tracing and screening in TB control programs. These strategies are essential for early detection and management of TB cases, preventing further transmission, and ensuring timely treatment. Effective contact tracing and rigorous screening are pivotal in controlling the spread of TB and achieving successful public health outcomes.

Limitations

The study had several limitations. As a hospital-based study, the findings may not be generalizable to the broader community. The relatively small sample size, shorter study duration, and the single-center design may limit the robustness of the conclusions. Mycobacterium tuberculosis detection was not confirmed with culture for MTB. Invalid and indeterminate results were excluded from the study. Future studies with larger, multicentre cohorts are needed to validate these findings.

## Conclusions

This study highlights the efficacy of CBNAAT in diagnosing PTB, especially in smear-negative cases, and underscores the ongoing challenge of rifampicin resistance. The findings emphasize the need for comprehensive TB control strategies that include rapid diagnostic tools, effective contact tracing, and addressing social determinants of health. Enhanced focus on nutrition and management of comorbidities will also be crucial in reducing the TB burden. Based on our findings, we recommend the continued use and expansion of CBNAAT for TB diagnosis, particularly in resource-limited settings. Public health interventions should focus on improving nutritional status and managing comorbid conditions among TB patients. Additionally, strengthening TB surveillance and contact tracing programs can help in early detection and management of drug-resistant TB cases.
